# Pathways and factors in calcium uptake through the skins of strawberry fruit

**DOI:** 10.1038/s41598-025-15749-4

**Published:** 2025-08-14

**Authors:** Grecia Hurtado, Simon Sitzenstock, Kristian Pineda, Moritz Knoche

**Affiliations:** Institute of Horticultural Production Systems, Leibniz University Hanover, Herrenhäuser Straße 2, 30419 Hannover, Germany

**Keywords:** Ca, Penetration, *Fragaria × ananassa*, Cuticle, Surfactant, Hygroscopicity, Developmental biology, Physiology, Plant sciences

## Abstract

High levels of fruit calcium (Ca) in strawberry are associated with low levels of surface disorders, including water soaking. Because of the dysfunctional xylem in developing strawberry, fruit Ca can only be increased by spray application. The objectives were to quantify ^45^Ca uptake through the fruit surface from drying spray droplets. Uptake of ^45^CaCl_2_ was initially rapid but slowed down after droplet drying. There was no difference in Ca uptake through the rim between achene depressions and that through the achene depressions. The uptake of ^45^CaCl_2_ increased with fruit development and with increasing spray concentration. Uptake was highest for CaCl_2_ and Ca(NO_3_)_2_, but was lower for organic salts. The surfactants Glucopon (0.01%) and Triton X-100 (0.1%) increased Ca uptake. Increased temperature and increased relative humidity increased Ca uptake. Incubating ripe strawberries for 1 h in deionized water increased microcracking. Despite the increased microcracking ^45^CaCl_2_ uptake did not increase. Spray applications of 30 mM in the greenhouse increased fruit Ca content and decreased susceptibility to water soaking. Our study establishes that strawberry fruit take up Ca very readily from surface sprays of either CaCl_2_ or Ca(NO_3_)_2_. Increases in fruit Ca decrease susceptibility to water soaking.

**Supplementary Information:**

The online version contains supplementary material available at 10.1038/s41598-025-15749-4.

## Introduction

Water soaking is an important surface disorder that causes substantial commercial loss in field-grown strawberry production, particularly in regions where rainfall occurs during the harvest season. The surface of a water-soaked strawberry fruit appears lighter in color and deliquescent. Water soaking impairs fruit quality both by compromising appearance and by increasing the incidence of fruit rots^1^. Calcium (Ca) is an important mineral governing quality in strawberry. Increased fruit Ca is associated with increased fruit firmness^2–4^ and decreased susceptibility to fruit surface disorders, such as water soaking^5^.

In most fruit crops, conventional applications of Ca salts to the soil surface are not effective in raising levels of Ca in the fruit. This is because Ca transport does not occur in the phloem, but only in the xylem, whereas the fruit xylem in many species suffers a progressive loss in functionality during development. This applies in cranberry^6^, plums^7^, cherries^8^, apples^9^ and strawberry^10^. Therefore, to increase fruit Ca content, the Ca must be applied directly to the fruit surface as a spray. For strawberry, there is only limited information on the effects of fruit-surface applications of Ca-salts on fruit Ca content and on fruit susceptibility to water soaking^5,11^. Moreover, the results of such studies are not always consistent^12–14^. The lack of consistency may result from a limited understanding of the physiology of Ca transport through the strawberry fruit skin. The naturally high variability in Ca content between individual fruit may be due to the effects of certain environmental factors on the rate of Ca penetration through the skin^15^.

The objectives of this study were: (1) to quantify Ca uptake through the strawberry fruit skin and (2) to identify the factors affecting uptake. In addition, we determined whether spray applications of un-labeled CaCl_2_ in the field, increased fruit Ca content and decreased susceptibility to water soaking. For the laboratory uptake studies, we used the radioactive tracer ^45^Ca that allows evaluation of uptake with high precision, even very low uptake can be detected accurately^16,17^. In contrast to our earlier study, the radioactive tracer allows to quantify Ca penetration from simulated spray droplets^11^. This technique mimics spray application in the field where spray droplets dry and the liquid phase of a spray droplet is typically short.

## Results

### Calcium uptake dynamics

**Fig. 1:**
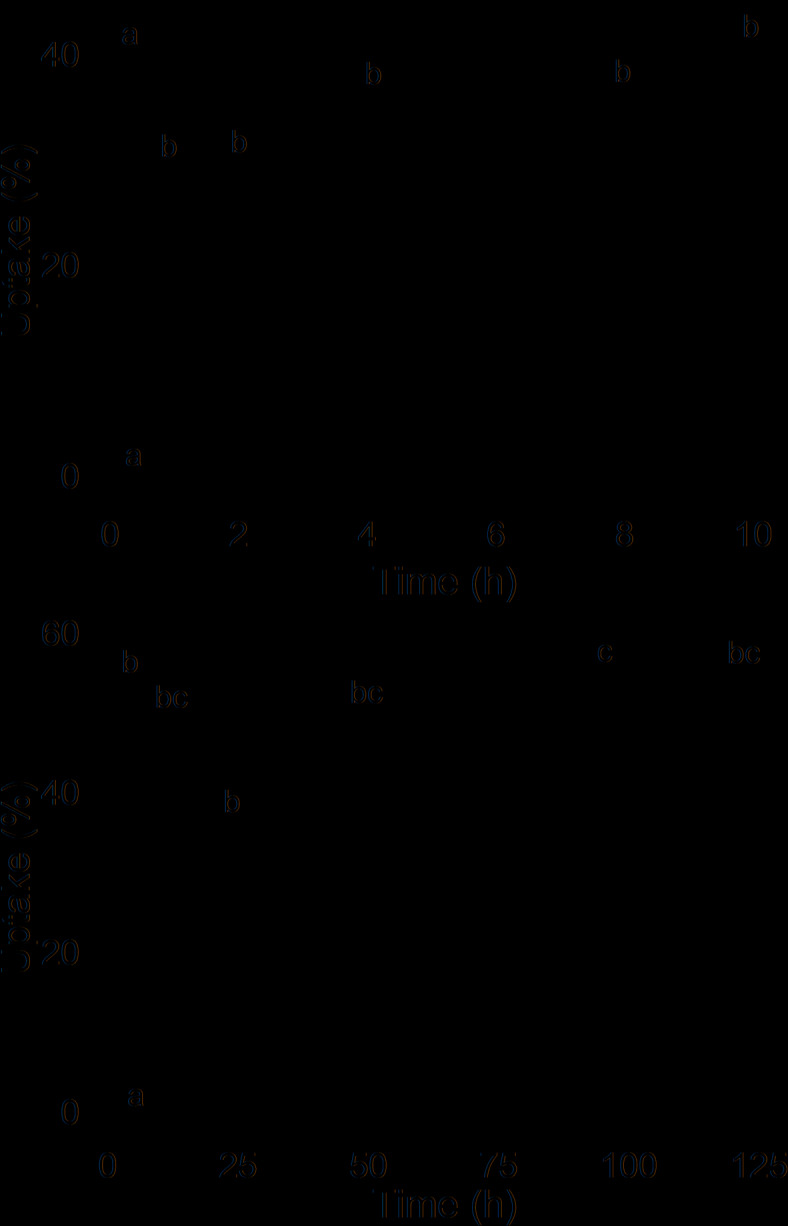
Short term (**a**) and long term (**b**) time courses of uptake of ^45^CaCl_2_ into ripe strawberry fruit. Data symbols followed by the same letter are not significantly different, Tukey’s studentized Range test, *p* = 0.05. The number of replications was ten.

Initially, penetration of ^45^CaCl_2_ into mature strawberries was rapid but then slowed down as time progressed (Fig. 1). About 53% of Ca uptake occurred in the first hour as the droplets dried. Strawberries have a corrugated surface comprised of achene depressions separated by rims. However, there was no difference in uptake regardless of whether a droplet was applied on the rim between adjacent achene depressions or in the achene depression itself (Table 1).

**Table 1:** ^45^Calcium uptake (%) in different sites of ripe cv. ‘Honeoye’ strawberry.

Site	Uptake (%)
Rim between depressions	32.4 ± 3.8^ns^
Achene depression	37.9 ± 5.5

^ns^Anova revealed no significant effect of site on ^45^Ca uptake. The number of replications was ten.

**Fig. 2:**
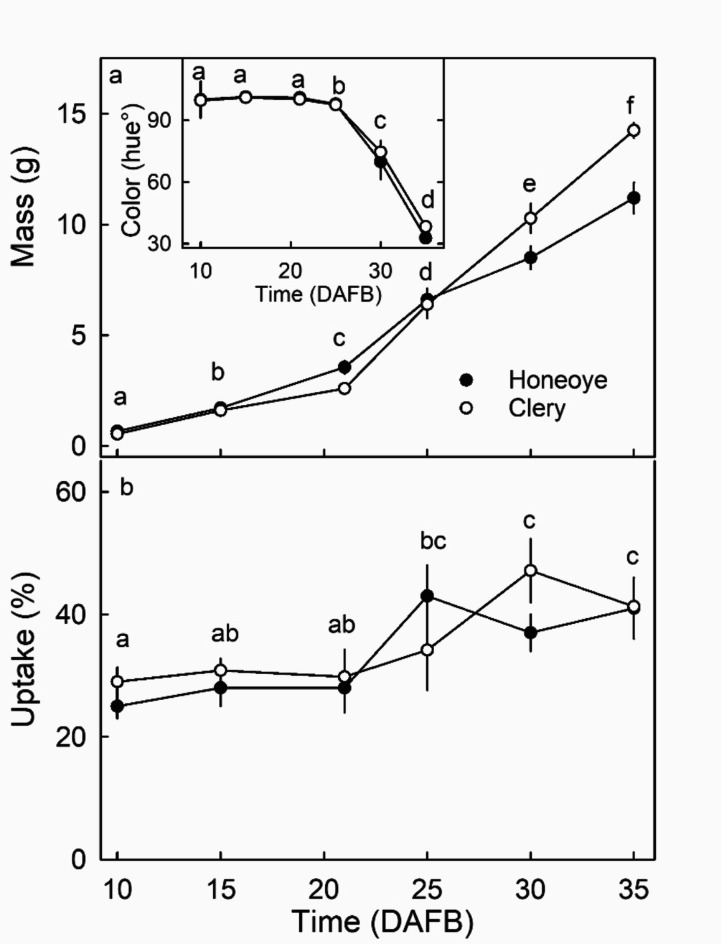
Developmental time course of change in mass (**a**), color (**a**, inset) and uptake of ^45^CaCl_2_ into ripe strawberry fruit (**b**). Significant main effect for ‘time’ but not ‘cultivar ‘ in two factorial analysis of variance. Data symbols followed by the same letter are not significantly different, pairwise Mann-Whitney U test at p = 0.05. The number of replications was ten.

Fruit mass increased during development (Fig. 2a). Color change from green to white began around 25 DAFB, fruit were fully red and ripe by 35 DAFB (Fig. 2, inset). Uptake of^ 45^CaCl_2_ increased with development and was highest at the stage when the fruit entered the red-ripe stage (Fig. 2b).

### Solution factors affecting calcium uptake

**Fig. 3:**
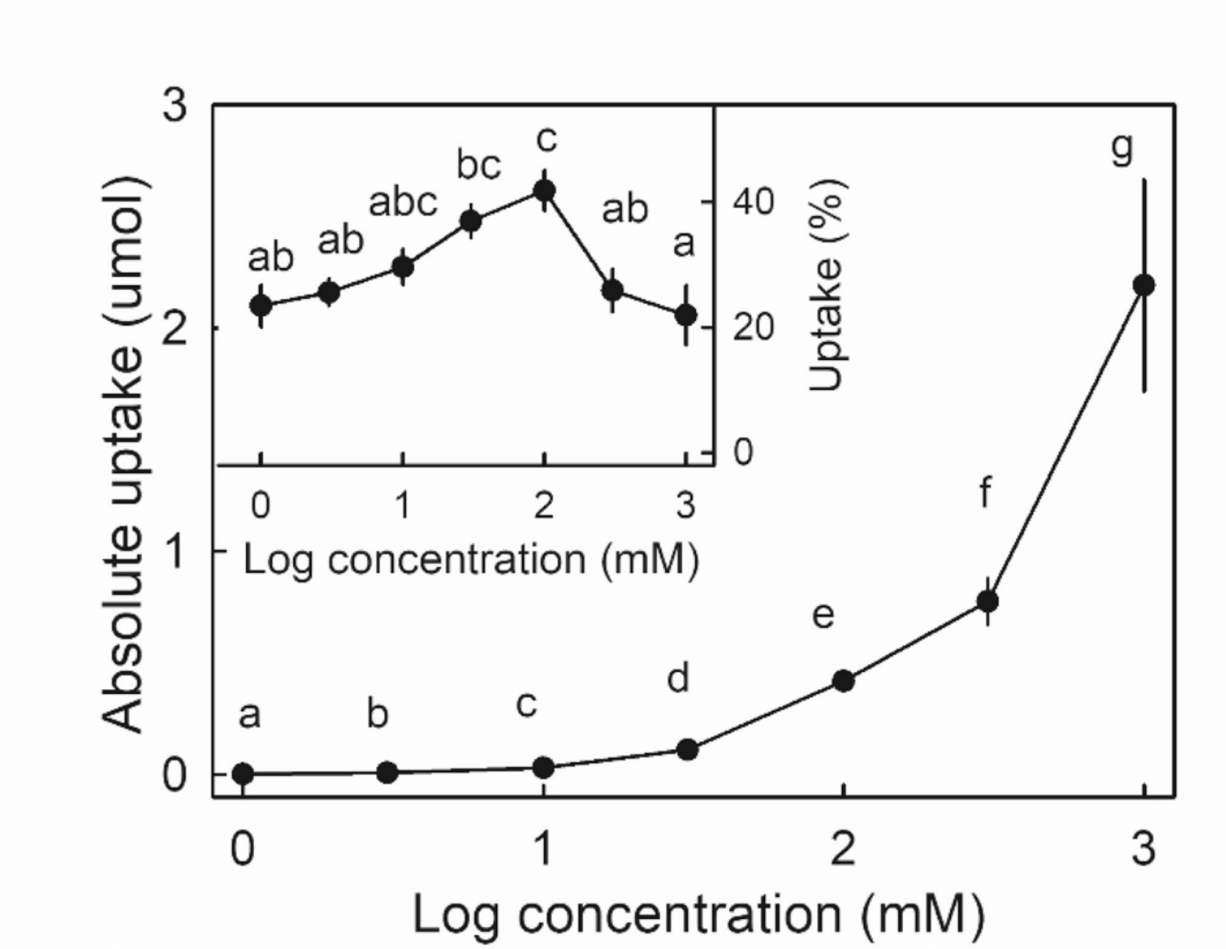
Effect of Ca concentration on total uptake of ^45^CaCl_2_ into ripe strawberry fruit. Inset: Effect of Ca concentration on ^45^CaCl_2_ uptake, expressed as a fraction (%) of the amount applied. Data symbols followed by the same letter are not significantly different, Tukey’s studentized Range test for % ^45^CaCl_2_ uptake, *p* = 0.05, pairwise Mann-Whitney U test for total uptake of ^45^CaCl_2_, *p* = 0.05. The number of replications was ten.

The absolute amount of ^45^CaCl_2_ taken up increased as the concentration applied increased (Fig. 3). On a percentage basis, however, uptake increased with concentration up to a maximum of 42% of the amount applied at 100 mM but then decreased at higher concentrations.

To maintain electric neutrality, uptake of the Ca cation is always accompanied by the corresponding anion(s). Uptake of Ca was highest from CaCl_2_ and Ca(NO_3_)_2_. Uptake of Ca was significantly lower for all salts with organic ion partners (Table 2). Organic Ca salts also had a higher molar mass and were less hygroscopic as indexed by the higher point of deliquescence (POD) than the inorganic CaCl_2_ and Ca(NO_3_)_2_. The POD represents the equilibrium relative humidity above which the droplet deposit remains liquid.

**Table 2:** Effect of calcium salt solutions (all at 30mM) on ^45^Ca uptake (%) into ripe cv. ‘Clery’ strawberries. The salts differed in molar mass and hygroscopicity. The hygroscopicity of the salt is characterized by the point of deliquescence (POD)^30^. The POD represents the equilibrium relative humidity above which the droplet deposit remains liquid.

Salt solution	Molar mass (g mol^− 1^)	POD^a^ (%)	Uptake (%)
CaCl_2_	111.0	33	40.5 ± 3.3 a*
Ca(NO_3_)_2_	164.1	56	32.7 ± 3.8 a
Ca-formate	130.1	–	8.6 ± 0.8 c
Ca-lactate	218.2	97	16.5 ± 2.7 b
Ca-heptagluconate	490.4	–	12.1 ± 1.2 b
Ca-propionate	186.2	95	14.1 ± 2.0 b
Ca-acetate	158.1	100	9.0 ± 0.8 c

*Mean separation within columns by Tukey’s studentized range test at *p* = 0.05. The number of replications was ten.

^a^POD data taken from^30^. There is no published data on the POD of Ca-formate and Ca-heptagluconate.

**Fig. 4:**
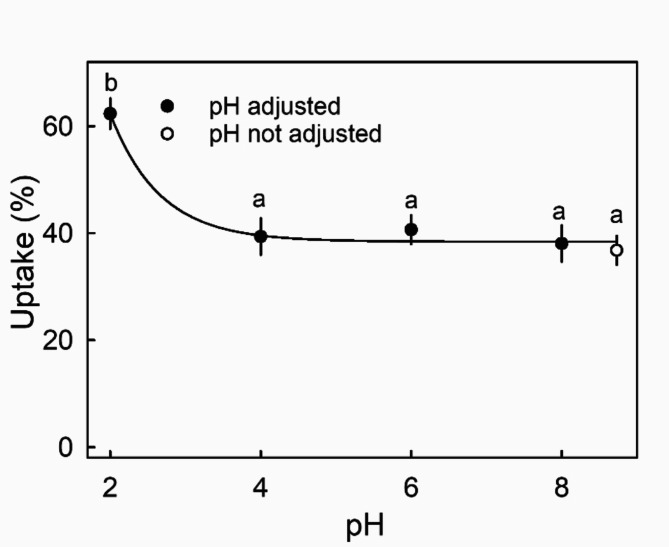
Effect of pH on uptake of ^45^CaCl_2_ into ripe strawberry fruit. Data symbols followed by the same letter are not significantly different, Tukey’s studentized Range test, *p* = 0.05. The number of replications was ten.

Cuticles carry a pH dependent charge that affects penetration of ions^18,19^. The pH of the CaCl_2_ solution had a marked effect on Ca uptake (Fig. 4). Uptake was highest at pH 2 – uptake was lower at all other pHs.

Addition of the surfactant Glucopon or Triton X-100 significantly increased uptake of CaCl_2_ (Table 3).

**Table 3:** Effect of surfactants on ^45^Ca uptake (%) into ripe cv. ‘Honeoye’ strawberries.

Surfactant	Uptake (%)
Control (water)	47.3 ± 3.6 b*
Glucopon	65.3 ± 2.6 a
Triton-X-100	64.7 ± 3.3 a

*Mean separation within columns by Tukey’s studentized range test at *p* = 0.05.

The number of replications was ten.

### Environmental factors affecting calcium uptake

**Fig. 5:**
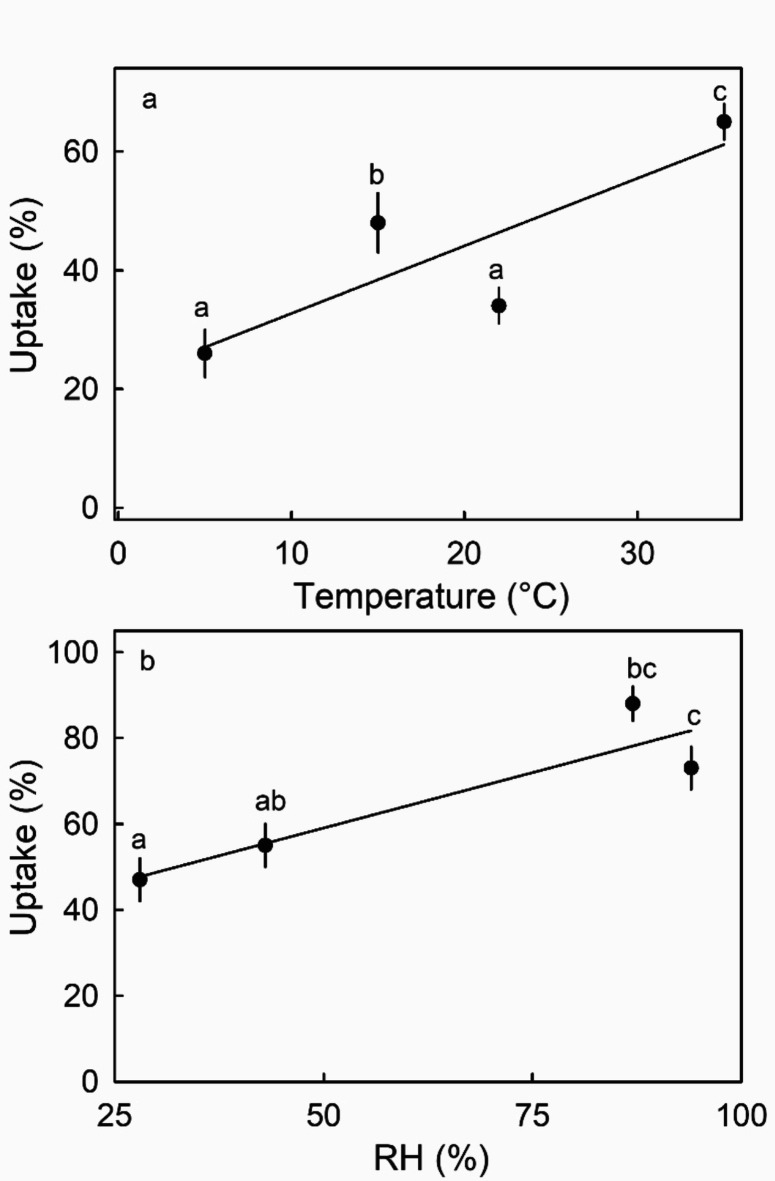
Effect of temperature (**a**) and relative humidity (**b**) on ^45^CaCl_2_ uptake into ripe strawberry fruit. Data symbols followed by the same letter are not significantly different, Tukey’s studentized Range test, *p* = 0.05. The number of replications was ten.

Increasing temperature or increasing RH increased Ca uptake (Fig. 5).

**Fig. 6:**
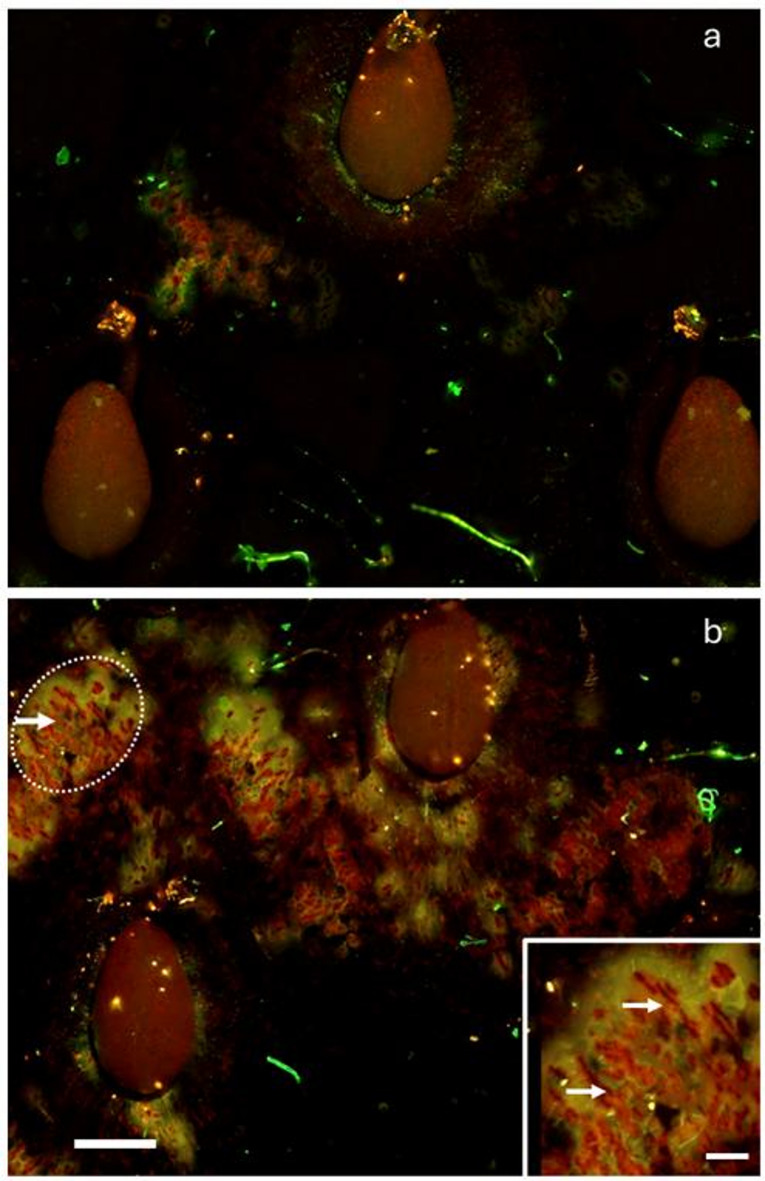
Fluorescent micrographs of the strawberry fruit surface before (**a**) and after induction (**b**) of microscopic cracks in the cuticle. White arrows identify microcracks. Microcracks are openings in the cuticle that allow the fluorescent tracer acridine orange to penetrate; orange, yellow, and green fluorescence denote decreasing tracer concentrations (see white arrows). (**b**, inset): Detail view of microcracks. Ripe strawberries were incubated for 1 h in deionized water to induce microcracks. Scale bar in main image: 500 μm and in Inset: 125 μm.

Simulating exposure of strawberries to rain by incubating ripe fruit for 1 h in deionized water markedly increased microcracking of the cuticle (Fig. 6). However, there was no significant increase in ^45^CaCl_2_ uptake despite significantly increased microcracking, as indexed by the percentage area infiltrated with acridine orange (Table 4).

**Table 4:** Effect of cuticular microcracks on ^45^Ca uptake into ripe cv. ‘Clery’ strawberries. Microcracking was induced by incubating strawberries in deionized water for 1 h. Fruit not incubated served as controls. Microcracking was indexed as the percentage of the fruit surface area infiltrated with the fluorescence tracer acridine orange. Acridine orange does not penetrate an intact fruit cuticle but does penetrate through imperfections such as microcracks.

Treatment	Microcracks	Uptake
	Infiltration area (%)	(%)
Control	10.3 ± 1.5 a*	38.9 ± 4.2 ^ns^
Microcracks	22.1 ± 2.2 b	47.6 ± 5.0

*Median separation within columns by pairwise Mann-Whitney U test at *p* = 0.05.

^(ns)^Means do not differ significantly. The number of replications was 60 for infiltration area and ten for uptake.

**Fig. 7:**
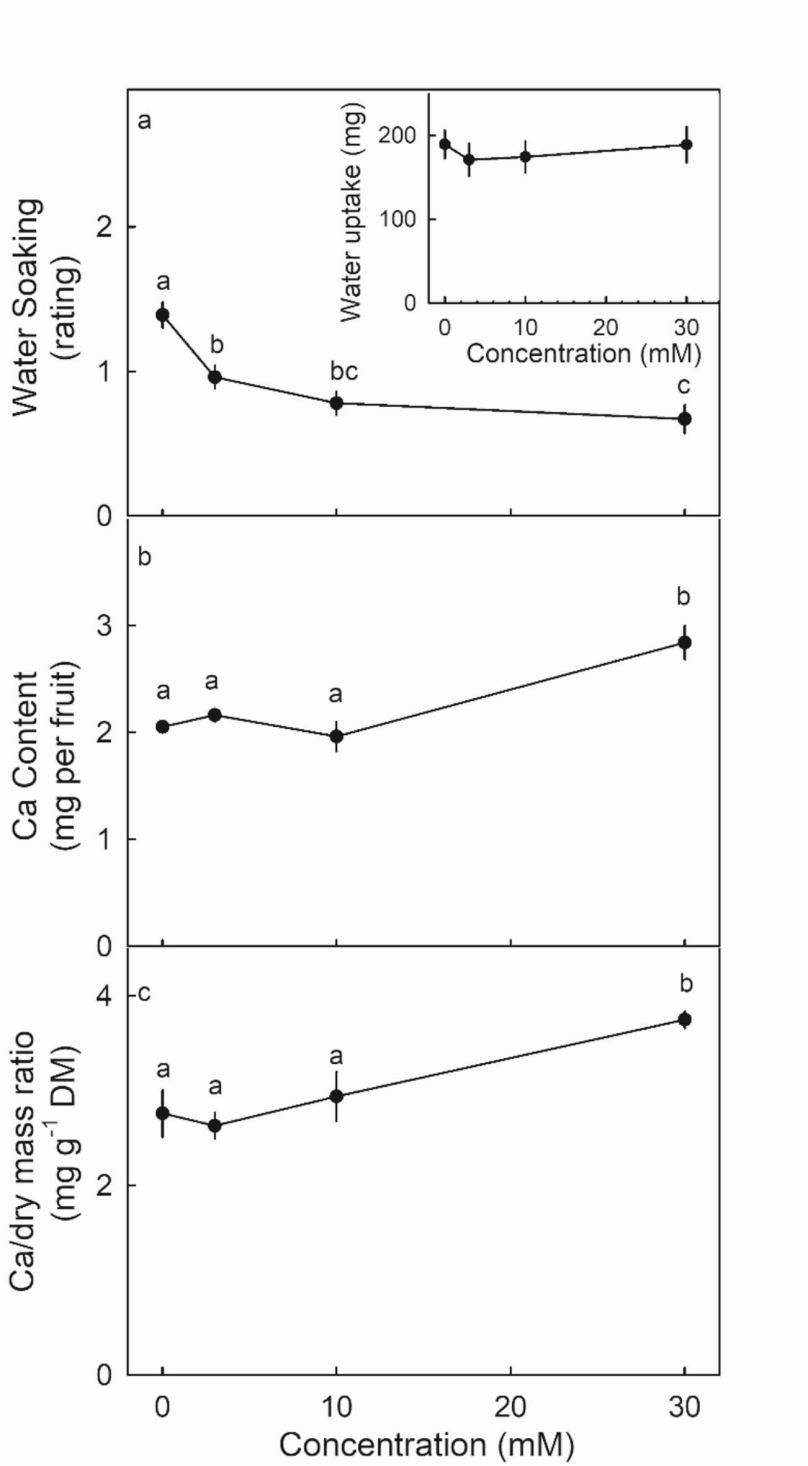
Effect of spay applications of CaCl_2_ at concentrations of 1, 3, 10 or 30 mM on the susceptibility of strawberries to water soaking (**a**), water uptake (**a**, inset), Ca content per fruit (**b**) and the Ca/dry mass ratio (**c**) of ripe strawberry fruit. Water soaking was indexed using a five-score rating scale: score 0, no water soaking; score 1, < 10% of the surface area water-soaked; score 2, 10 to 35%; score 3, 35 to 60%; score 4 > 60%. Data symbols followed by the same letter are not significantly different, pairwise Mann-Whitney U test at *p* = 0.05 for water soaking and Ca/dry mass ratio and Tukey’s studentized range test at *p* = 0.05 for Ca content. Differences in water uptake not significant. For Ca-measurement 8 groups of 10 fruit each were used and for water soaking and water uptake a set of 80 individual fruit was used for each treatment.

Spraying potted plants with CaCl_2_ solution decreased susceptibility to water soaking and did not affect water uptake (Fig. 7a). Plants sprayed with 30 mM CaCl_2_, showed significant increases in Ca content and in Ca/dry mass ratio (Fig. 7b, c).

## Discussion

The main finding was that Ca uptake through the strawberry fruit skin is especially rapid before the droplet dried and slowed considerably after drying from the solid residue. Uptake of Ca would seem to be determined by the mobility of Ca in solid deposit. This finding is new.

In our subsequent discussion we focus on (1) a comparison of experimental systems used for determining Ca uptake, (2) the characteristics of Ca salts that affect uptake, and (3) pathways for Ca uptake through the strawberry fruit skin.

### Comparing experimental systems for studying Ca uptake

The experimental methods used here differ from the incubation assays used in earlier studies on Ca uptake into strawberries^11,20^. As with spray applications in the field, Ca was applied to the fruit surface as a (simulated) spray droplet that is subject to rapid drying. The footprint of the initial droplet represents the cross-sectional area for Ca uptake before the droplet dries. On drying, the footprint of the solid residue is typically smaller than this. During drying, the Ca concentration of the droplet and, hence, the concentration gradient driving diffusional penetration, increases continuously. Thus, penetration occurs under non-steady state (changing) conditions. These conditions mimic those in the field for spray applications. Since droplet number and droplet volume were small and the fraction of the Ca salt taken up was high (c. 40%), our system resembled a ‘finite dose system’ often used in cuticular penetration studies^16,17,21^. Typically, radiolabeled Ca salts are used to detect even small amounts of Ca uptake relative to the highly variable background levels of Ca of strawberry fruit^15^.

In contrast, in the incubation assays cited above, fruit was incubated by immersion in dilute solutions of Ca salts. Hence, the volumes of the incubation solutions were several-times larger than the volumes of the incubated fruit. Due to the low uptake, the concentration gradient of Ca across the fruit skin remained approximately constant throughout the experiment. Moreover, the cross-sectional area through which Ca uptake occurred was also constant, being equal to the fruit surface area. Consequently, these Ca uptakes took place under steady state conditions in a well-defined system. These conditions resemble ‘infinite dose systems’ commonly used in studies of cuticular penetration of agrochemicals^16,17^. However, the use of a radiolabeled Ca salt in a whole-fruit incubation assay would be prohibited by the safety regulations, so only un-labeled Ca salts can be used in this way. Furthermore, quantification of uptake requires laborious sample processing to determine the fruit Ca content or the Ca/dry mass ratio^11,15^. Because fruit Ca content is a highly variable parameter^15 ^Ca uptakes must greatly exceed the natural Ca levels to be detectable against this high background noise. This has not always been the case^11^.

### Characteristics of Ca salts that affect uptake from drying droplets

Common to both types of assay are the characteristics of the Ca salts (primarily polarity and molecular size). In addition, in finite dose assays, once the droplet has dried, the characteristics of solid deposit are critical. In the field, drying usually occurs within minutes of spray application (depending on the meteorological conditions at the time) whereas in the laboratory drying takes about an hour because much larger droplets were used. Efficient penetration of Ca from the deposit can occur only if the concentration gradient between the deposit and the fruit skin tissues is high. It is this concentration gradient that represents the diffusional driving force for penetration during and after droplet drying. The results obtained with our ‘finite dose system’ demonstrate very limited penetration occurs from this solid deposit. This conclusion is based on the following observations.

First, most Ca penetration occurred during the first hour after application, and before the droplet dried. Very little penetration occurred thereafter.

Second, high RH increased CaCl_2_ penetration. The high RH likely slowed drying so the droplet (liquid phase Ca) remained in contact for longer, compared with under low RH conditions. Also, a high humidity generated a high moisture content of the deposit. Similar findings were reported in earlier studies for penetration of plant growth regulators and CaCl_2_ in other fruit crops^22–24^.

Third, uptake was higher for those Ca salts that had low points of deliquescence (POD). The POD represents the equilibrium humidity above which a seemingly dry droplet residue remains hydrated and hence maintains a high driving force for penetration. Only when the humidity falls below the POD will the Ca-salt form a completely dry deposit, such that the driving force falls away^25^. This explains the consistently low uptake of Ca from the organic Ca salts that all have low PODs^26^. It also accounts for the effects of salts on penetration of polar plant growth regulators such as aminoethoxyvinylglycine (AVG). Adding LiCl (POD 11%) and CaCl_2_ (POD 28%) at 10 mM to the simulated spray solution markedly enhanced AVG penetration.

Fourth, surfactants significantly increased Ca uptake. Surfactants affect uptake of agrochemicals and foliar fertilizers in multiple ways including (1) by increasing the contact area between the droplet or the deposit and fruit surface via a decrease in surface tension^27^ (2) by generating a hydrated deposit^28^ and (3) by increasing the permeability of the cuticle^29,30^.

The strawberry fruit cuticle is markedly strained^31^ and exposure of the strained cuticle on the fruit surface to water or high humidity causes microcracking of the cuticle^32^.

Whether hygroscopic spray deposits aggravate microcracking of the strained strawberry fruit cuticle or increase the risk of microbial infections, is currently unknown. Both aspects deserve further study.

### Pathways for Ca uptake through the strawberry skin

Important determinants of Ca penetration are high-flux pathways resulting from polar pathways and cuticular microcracks.

*Polar pathways* - Skin penetration along polar pathways is likely to be enhanced by a more hydrated deposit. For a number of fruit crops, including strawberries, studies have established that a liquid continuum exists across a hydrated cuticle^33–36^. This continuum allows passage of small, polar molecules across a lipophilic cuticle^34,36^. This continuum is referred to as a polar pathway^29,35–38^. Polar pathways are the result of the specific orientation of polar functional groups in a hydrated cuticle. Penetration along a polar pathway is rapid and also size-selective. The molecular sizes of the Ca salts investigated here were all below the size exclusion limit for the strawberry fruit cuticle that has been estimated at about 1500 g mol^− 1 34^.

*Cuticular microcracks* – Another high flux pathway for uptake are the microcracks that have frequently been observed on the strawberry fruit surface, and also on many other fruit crop species^32,39^. Microcracks result from (1) an overly strained cuticle, which is a consequence of the cessation of cuticular deposition early on in fruit development, with significant fruit surface expansion occurring thereafter^31^ and (2) exposure of the strained cuticle to high aerial humidities or to the presence of liquid water^32^. In addition, the complex topography of the strawberry fruit surface causes stress concentrations and microcracking, particularly around the achene depressions^40^. These depressions occur because the achenes are connected to the vascular system. This connection is rigid and so restricts expansion growth. Consequently, depressions form at achenes while the area surrounding the achenes expands with fruit growth and forms ridges between the achene depressions. This growth pattern causes stress concentration that leads to cuticular microcracking in this and our earlier study^32^. Microcracks impair the barrier functions of the cuticle, so making it more permeable to water and less effective in excluding pathogens. Penetrants, such as Ca, can bypass the cuticle barrier by entering the fruit via cuticular microcracks. This likely also occurs in strawberry with Ca salts. That the effect of microcracking on Ca uptake was not significant in our study may have resulted from (1) the small area covered by the spray droplets (footprint ~ 13 mm^2^ and the probability of hitting a microcrack and (2) the high variability (both from fruit: fruit and from region: region on a fruit (~ 15% fruit surface microcracked)) of microcracking, as indicated by the large standard errors.

### Practical implications

Sprays of 30 mM CaCl_2_ significantly increase fruit Ca content (by about 40%) and reduce water-soaking susceptibility (by about 50%). The effect of Ca on water soaking probably results from multiple factors including: a reduction in microcracking of the cuticle, decreased leakage of the plasma membrane and, probably, increased crosslinking of cell wall constituents^5^. Calcium uptake through the fruit skin depends on the stage of development. As in apple fruit, the skins of young strawberries are more permeable than those of older ones^41^. However, young strawberries are small and hence, represent a more difficult-to-hit target. Hence, to maximize the increase fruit Ca, repeated applications of Ca-salts are required to decrease the susceptibility to water soaking. Incidentally, a CaCl_2_ spray of higher concentration than about 30 mM tends to be phytotoxic, so sub-toxic concentrations must be used. Our research also reveals that the mobility of Ca in the droplet deposit may limit penetration. Of the salts investigated CaCl_2_ and Ca(NO_3_)_2_ are the most readily taken up by strawberries. Adding a surfactant such as Glucopon or TX-100 further increases uptake, probably by increasing the area of the droplet footprint, by increasing cuticle permeability and by maintaining a more hydrated droplet deposit. The effect of these combinations on decreasing the susceptibility to water soaking should now be tested under field conditions.

## Materials and methods

### Plant material

Strawberry fruits (*Fragaria × ananassa* Duch.) of cvs. ‘Clery’ and ‘Honeoye’ were grown in a greenhouse or a growth chamber on the Herrenhausen Campus of Leibniz University, Hannover, Germany (lat. 52°23′N, long. 9°42′E). In the latter, day/night temperature and relative humidity (RH) were set at 20/16°C and 60/75% RH during a 16 h day/night photoperiod. Plants were grown in pots (14 cm diam.) filled with a peat-moss based growing medium (Einheitserde Pikiererde Typ P; Einheitserde Werkverband e.V., Sinntal-Altengronau, Germany).

Fruits were harvested at commercial ripeness (~ 80% of surface area red^42^) unless specified otherwise. Fruits were selected for uniformity of size, shape, color and the absence of visual defects.

### General procedure for ^45^Ca uptake experiments

Fruits were harvested by cutting the peduncle under water to avoid embolism of the vascular system. Fruit were immediately placed on a six-well plate filled with deionized water such that the peduncle extended through a hole in the lid into the water. A soft foam ring was placed between the lid and the fruit to support the fruit without causing mechanical damage. A maximum of four fruits were placed per plate.

A solution of 30 mM of CaCl_2_ was spiked with ^45^CaCl_2_ (specific activity 1.1 GBq mg^− 1^; PerkinElmer, Waltham, MA, USA). The radioactive concentration of the treatment solution averaged about 855 dpm µl^− 1^. Ten 1 µl droplets were applied to the surface of a strawberry using a microliter syringe equipped with a manual dispenser (Hamilton Bonaduz, Bonaduz, Switzerland). Working on one side of the fruit, unless specified otherwise, half of the droplets were applied to the rim between achene depressions and the other half into the achene depression. The droplets were allowed to dry. The treated area was marked with a permanent marker. Unless specified otherwise, droplet residues were later removed by rinsing the treated fruit surface with 5 ml of 50 mM citric acid. Preliminary experiments established that 5 ml of citric acid was most effective for removing surface residues of ^45^CaCl_2_ from the topographically irregular surface of a strawberry fruit (Table S1 and Fig. S1). A 1 ml aliquot of the rinse was sampled. The fruit was dried in an oven at 50 °C, then transferred into a 20 ml glass scintillation vial, and ashed at 500 °C (heated from 22 to 500 °C for 2 h, then held at 500 °C for 4 h) in a muffle furnace (L24/11/B180; Nabertherm, Lilienthal, Germany). When ashing was incomplete, as indicated by black residues in the sample, a few droplets of 1 M HCl were added, and the sample re-ashed. The ash was taken up in 1 ml of 1 M HCl and 10 ml of scintillation cocktail (Ultima GoldTM XR; Perkin-Elmer Life and Analytical Sciences, Boston, MA, USA). The radioactivities of the surface rinse and of the ash were measured by liquid scintillation spectrometry (LS 6500; Beckman Instruments, Fullerton, CA, USA). The percentage of uptake was calculated from the radioactivity measured in the ash, divided by the radioactivity applied per fruit multiplied by 100 (%). The total recovery of radioactivity was calculated by summing the radioactivity in the rinse and in the ash fractions and dividing by the radioactivity applied per fruit multiplied by 100 (%). The recovery of radioactivity was always greater than 93%. The total number of individual fruit replicates was ten.

### Experiments

In the following experiments, ^45^Ca uptake was measured using the general procedure described above.

### Calcium uptake dynamics

*Time courses* - Short- and long-term time courses of Ca uptake were established in cv. ‘Clery’ strawberries. For the short-term ones, the fruit were sampled at 0, 1, 2, 4, 8 and 12 h, and for the long-term ones at 0, 12, 24, 48, 96 and 120 h.

*Achene depression vs. rim* - Strawberries have a complex fruit surface comprised of achene depressions separated by rims^32^. Potential differences in Ca uptake in the rim between achene depressions and in the achene depressions were established by applying 10 droplets of 1 µl either on the rims between achene depressions or in the achene depressions of cv. ‘Honeoye’ strawberries.

*Developmental time course* - The effect of the development time course on Ca uptake was studied in both cv. ‘Clery’ and cv. ‘Honeoye’. Fruit were treated at 10, 15, 20, 25, 30 or 35 days after full bloom (DAFB). For fruit younger than 20 DAFB differentiation between rim and achene depression was not possible for droplet application. Fruit mass and color (CM-2600 d, Konica Minolta, Tokyo, Japan) were measured on fruit from the same batch.

### Solution factors

*CaCl*_*2*_ *concentration* - The effect of CaCl_2_ concentration on Ca uptake was determined in cv. ‘Clery’. The concentrations used were 1, 3, 10, 30, 100, 300 or 1000 mM CaCl2.

*Anions* - To maintain electric neutrality, anion penetration must accompany penetration of the Ca cation. The effect of the partner anions on Ca uptake was investigated using cv. ‘Clery’ strawberries. Solutions of Ca-formate, Ca-lactate, Ca-heptagluconate, Ca-propionate, Ca-acetate, Ca(NO_3_)_2_ and CaCl_2_ (all at 30 mM) were spiked with ^45^CaCl_2_ (1.1 GBq mg^− 1^; PerkinElmer, Waltham, MA, USA) and applied as described above.

*pH* - Cuticles are polyelectrolytes that carry charges depending on pH^18,19^. These charges may affect penetration of ions. The effect of treatment solution pH on Ca uptake was studied in cv. ‘Clery’. The pHs of the 30 mM CaCl_2_ solutions were adjusted by titrating with 1 M HCl to pH values of 2, 4, 6 or 8. The volumes of HCl added to 50 ml of a 30 mM CaCl_2_ solution for pH adjustment to pH 2, 4, 6 or 8 were 530.0, 16.5, 7.5–0.7 µl, respectively.

*Surfactants* - The effects of surfactants on Ca uptake were studied using the surfactants Glucopon 215 UP (BASF, Ludwigshafen, Germany) at 0.01% (w/v) or Triton-X-100 (Union Carbide, Antwerpen, Belgium) at 0.1% (w/v). A solution without surfactant was used as control. Treatments were applied to cv. ‘Honeoye’ strawberries as described above.

### Environmental factors

*Temperature* - The effects of temperature on Ca uptake was studied at 5, 15, 22, or 35 °C on cv. ‘Honeoye’ strawberries.

*Relative humidity* - The effects of RH on Ca uptake were determined by holding cv. ‘Honeoye’ fruit in a closed polyethylene box above dry silica gel or above slurries of CaCl_2_, NaCl or KNO_3_. The equilibrium RHs above these are 28, 43, 87 or 94%, respectively^43^.

*Microcracking* - Exposure of the strawberry fruit surface to surface water or high humidity results in microcracking of the strained cuticle^32^. The effects of microcracking of the cuticle of ripe cv. ‘Clery’ strawberries on ^45^Ca uptake were investigated by inducing microcracking using the procedure described earlier^32^. Briefly, a fruit was completely submerged in deionized water for 1 h using a soft foam plug. After the induction period, the fruit was transferred to a 6-well plate. A set of un-incubated fruits was used as control. Droplets of the ^45^CaCl_2_ solution were applied as described above. On a second set of fruits from the same batch, the extent of microcracking after 1 h of water induction was assessed using the procedure of Peschel and Knoche^39^. Briefly, a fruit was incubated in 0.1% (w/w) acridine orange (Carl Roth, Karlsruhe, Germany) for 5 min, then rinsed thoroughly with deionized water. The fruit skin was then inspected by fluorescence microscopy (MZ10F; Leica Microsystems, Wetzlar, Germany). Four calibrated images were taken (Camera DP73; GFP-plus filter, 480/40 nm excitation, ≥ 510 nm emission wavelength). The infiltrated area was measured using image analysis (cellSens Dimension 1.16 Desktop; Olympus Soft Imaging Solutions, Münster, Germany). Microcracking of the cuticle was quantified as the percentage of the area in the microscope window, found to be infiltrated with acridine orange^32^. A tissue infiltrated with acridine orange exhibits orange, yellow and green fluorescence^39^. The total number of replications was 60.

### Ca uptake by spray application

The effect of CaCl_2_ concentration on fruit Ca content was investigated by spraying fruit with 0, 3, 10 or 30 mM CaCl_2_. Potted strawberry plants were sprayed weekly during the entire development period, beginning at 2–3 DAFB and continuing till ripeness. At least four sprays were applied in the morning (between 8:00–9:00 a.m.) until all fruitlets were completely wet. A plastic shield was inserted during spraying to prevent spray deposition on the soil surface and so potential Ca-uptake via the roots. The fruit were harvested when ripe and their Ca-contents were quantified with four replicates of ten fruit each using the procedure described earlier^44^. Briefly, a fruit was weighed to determine fresh mass, freeze-dried for 3 to 4 d, then dried at 103 °C for 15 d, weighed to determine dry mass and then ground in a ball mill (MM 400 mill; Retsch, Haan, Germany). The ground powder was re-dried at 103 °C for at least 3 to 4 days and an aliquot of 100 mg ashed in a muffle furnace (L24/11/B180; Nabertherm, Lilienthal, Germany). The ash was taken up in 2 ml of 1 M HCl and 8 ml deionized water and then filtered (MN 640 m; Macherey-Nagel, Düren, Germany). To avoid interference with phosphorus (P) in Ca measurement by atomic absorption spectroscopy (AAS), 1% of lanthanum chloride (LaCl_3_) was added to the solutions^45^. Solutions were analyzed by AAS (1100B; Perkin Elmer, Shelton, USA) equipped with a hollow cathode lamp for Ca-Mg using an air–acetylene flame.

An additional 40 fruit were harvested and incubated individually in deionized water to induce water-soaking. The fruit was held under the water using a soft plastic foam plug. After 4 h fruit were removed from the solution and carefully blotted using soft tissue paper. Water uptake was quantified gravimetrically by recording fruit weight before and after incubation. Water soaking was assessed using a five-point rating scale based on the % of the area of the surface water-soaked. The rating scale was: score 0 = no water soaking; score 1 < 10%; score 2 = 10 to < 35%; score 3 = 35 to 60% and score 4 > 60% of the surface area water-soaked^1^. The whole experiment was repeated twice.

### Data analyses

All experiments were conducted and analyzed using completely randomized designs. The only exception was the spray application experiment that was conducted using a randomized block design with four blocks of 10 plants per treatment. Data are presented as means ± standard errors. Data were analyzed by analysis of variance or – when the assumptions of normality or homogeneity of variance were violated - the non-parametric Kruskal-Wallis’s test. Means were compared using Tukey’s studentized range tests, and the pairwise Mann-Whitney U test at *p* = 0.05 using R studio (R version 4.1.0; R Foundation for Statistical Computing, Vienna, Austria).

## Supplementary Information

Below is the link to the electronic supplementary material.

Supplementary Material 1

Supplementary Material 2

## Data Availability

All data generated or analyzed during this study are included in this published article and its supplementary data file.
