# Supplementary material for: Pathways and factors in calcium uptake through the skins of strawberry fruit
Source: Sci Rep. 2025 Aug 14;15:29840. doi: 10.1038/s41598-025-15749-4 (PMC12354678; doi:10.1038/s41598-025-15749-4)
Supplement: Supplementary file 2 — Supplementary Material 2 [file 41598_2025_15749_MOESM2_ESM.docx]

**Supplementary data**

**Table S1.** Rinsing recovery from 10 x 1 µl of 30 mM 45CaCl2 applied on a cover slide after 2 h of drying in a sealed plastic box with dry silica gel (~0% relative humidity).

| Rinsing solution | Rinsing volume (ml) | Radioactivity in rinse (% of applied) |
| --- | --- | --- |
| Deionized water | 2 | 91.3 ± 1.0 |
| Ethanol 50% v/v | 2 | 76.6 ±3.0 |
| Aceton 50% v/v | 2 | 85.4 ±1.0 |
| Citric acid 50 mM | 2 | 93.8 ±1.8 |
| Malic acid 50 mM | 2 | 90.4 ±2.0 |


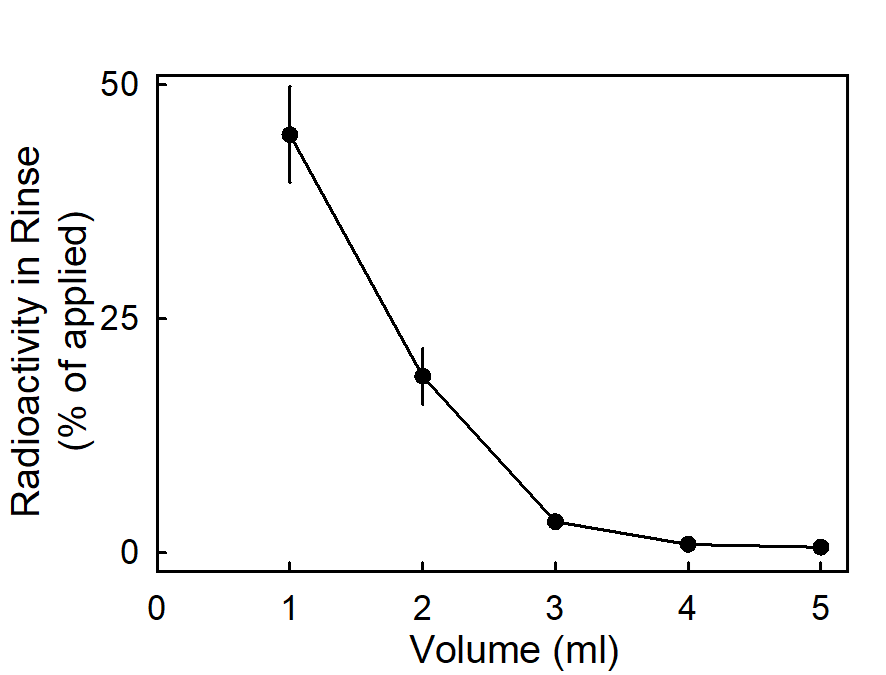


**Fig S1.** Recovery after sequential rinses, each with 1 ml of 50 mM citric acid, to remove surface residues of ^45^Cacl2 on strawberry fruit. The fruit content accounted for 28.8% of the total activity applied. The rinse contents plus the fruit contents indicates a 97.1% recovery of the total radioactivity applied.
